# Supplementary material for: Seasonal Arctic sea ice forecasting with probabilistic deep learning
Source: Nat Commun. 2021 Aug 26;12:5124. doi: 10.1038/s41467-021-25257-4 (PMC8390499; doi:10.1038/s41467-021-25257-4)
Supplement: Supplementary file 1 — Supplementary Information for Seasonal Arctic sea ice forecasting with probabilistic deep learning [file 41467_2021_25257_MOESM1_ESM.pdf]

Supplementary Information for

Seasonal Arctic sea ice forecasting with probabilistic deep learning

Tom R. Andersson[1*], J. Scott Hosking[1,2], María Pérez-Ortiz[3], Brooks Paige[2,3], Andrew Elliott[2,4], Chris Russell[5], Stephen Law[2,6], Daniel C. Jones[1], Jeremy Wilkinson[1], Tony Phillips[1], James Byrne[1], Steffen Tietsche[7], Beena Balan Sarojini[7], Eduardo Blanchard-Wrigglesworth[8], Yevgeny Aksenov[9], Rod Downie[10], and Emily Shuckburgh[1,11]

[1]British Antarctic Survey, NERC, UKRI, Cambridge, UK. [2]The Alan Turing Institute, London, UK. [3]Department of Computer Science, University College London, London, UK. [4]School of Mathematics and Statistics, University of Glasgow, Glasgow, UK. [5]Amazon Web Services, Tübingen, Germany. [6]Department of Geography, University College London, London, UK. [7]European Centre for Medium-Range Weather Forecasts (ECMWF), Reading, UK. [8]Department of Atmospheric Sciences, University of Washington, Seattle, WA, USA. [9]National Oceanography Centre, Southampton, UK. [10]WWF, Woking, UK. [11]University of Cambridge, Cambridge, UK.

*Correspondence: tomand@bas.ac.uk

Input Layer #	Layer #	Layer type	Activation	Resample	Norm	Output Shape
-	1	Input data	-	-	-	432 x 432 x 50
1	2	Conv 3 x 3	ReLU	-	-	432 x 432 x 128
2	3	Conv 3 x 3	ReLU	2 x 2 Downsample	BatchNorm	216 x 216 x 128
3	4	Conv 3 x 3	ReLU	-	-	216 x 216 x 256
4	5	Conv 3 x 3	ReLU	2 x 2 Downsample	BatchNorm	108 x 108 x 256
5	6	Conv 3 x 3	ReLU	-	-	108 x 108 x 512
6	7	Conv 3 x 3	ReLU	2 x 2 Downsample	BatchNorm	54 x 54 x 512
7	8	Conv 3 x 3	ReLU	-	-	54 x 54 x 512
8	9	Conv 3 x 3	ReLU	2 x 2 Downsample	BatchNorm	27 x 27 x 512
9	10	Conv 3 x 3	ReLU	-	-	27 x 27 x 1024
10	11	Conv 3 x 3	ReLU	2 x 2 Upsample	BatchNorm	54 x 54 x 1024
11	12	Conv 3 x 3	ReLU	-	-	54 x 54 x 512
9	13	Concat	-	-	-	54 x 54 x 1024
13	14	Conv 3 x 3	ReLU	-	-	54 x 54 x 512
14	15	Conv 3 x 3	ReLU	2 x 2 Upsample	BatchNorm	108 x 108 x 512
15	16	Conv 3 x 3	ReLU	-	-	108 x 108 x 512
7	17	Concat	-	-	-	108 x 108 x 1024
17	18	Conv 3 x 3	ReLU	-	-	108 x 108 x 512
18	19	Conv 3 x 3	ReLU	2 x 2 Upsample	BatchNorm	216 x 216 x 512
19	20	Conv 3 x 3	ReLU	-	-	216 x 216 x 256
5	21	Concat	-	-	-	216 x 216 x 512
21	22	Conv 3 x 3	ReLU	-	-	216 x 216 x 256
22	23	Conv 3 x 3	ReLU	2 x 2 Upsample	BatchNorm	432 x 432 x 256
23	24	Conv 3 x 3	ReLU	-	-	432 x 432 x 128
3	25	Concat	-	-	-	432 x 432 x 256
25	26	Conv 3 x 3	ReLU	-	-	432 x 432 x 128
26	27	Conv 3 x 3	ReLU	-	-	432 x 432 x 128
27	28	Conv 3 x 3	ReLU	-	-	432 x 432 x 128
28	29	Conv 3 x 3	Linear	-	-	432 x 432 x 3
28	30	Conv 3 x 3	Linear	-	-	432 x 432 x 3
28	31	Conv 3 x 3	Linear	-	-	432 x 432 x 3
28	32	Conv 3 x 3	Linear	-	-	432 x 432 x 3
28	33	Conv 3 x 3	Linear	-	-	432 x 432 x 3
28	34	Conv 3 x 3	Linear	-	-	432 x 432 x 3
29-34	35	Stack	-	-	-	432 x 432 x 3 x 6
35	36	Temp scale	-	-	-	432 x 432 x 3 x 6
36	37	Output	Softmax	-	-	432 x 432 x 3 x 6

Supplementary Table 1 | **IceNet's U-Net architecture and connections between layers.** Horizontal lines segment individual convolutional blocks. Layers 2-9 form the downsampling encoding path, layers 10-11 form the bottleneck, and layers 12-28 form the upsampling decoding path. Layers 29-37 are IceNet's custom output path, relating to the 6 forecast months and 3 output classes with the temperature scaling step. The inputs to the concatenation layers in the decoding path occur before the downsample operation.

Input variable name	Lead or lag (months)	Source
Linear trend forecast	1-6	Computed from OSI-SAF
SIC	1-12	OSI-SAF
2-metre air temperature anomaly	1-3	ERA5
500 hPa air temperature anomaly	1-3	ERA5
Sea surface temperature anomaly	1-3	ERA5
Downwards surface solar radiation anomaly	1-3	ERA5
Upwards surface solar radiation anomaly	1-3	ERA5
Sea level pressure anomaly	1-3	ERA5
500 hPa geopotential height anomaly	1-3	ERA5
250 hPa geopotential height anomaly	1-3	ERA5
10 hPa zonal wind speed	1-3	ERA5
10-metre X-direction wind speed	1	ERA5
10-metre Y-direction wind speed	1	ERA5
Land mask	N/A	OSI-SAF
Cosine of initialisation month index	N/A	N/A
Sine of initialisation month index	N/A	N/A

Supplementary Table 2 | **IceNet's input variables and their sources for the observational training dataset.**

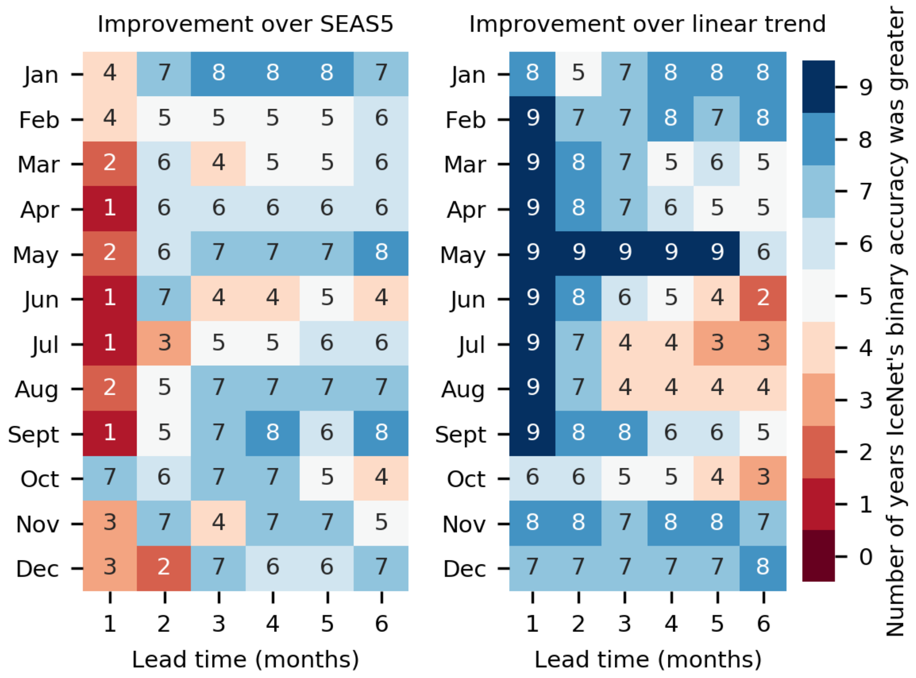

Supplementary Fig. 1 | **Consistency of IceNet's performance** . Number of years that IceNet's binary accuracy exceeded that of SEAS5 (left) and the linear trend (right) across the nine validation and test years (2012-2020), shown for each forecast calendar month and lead time. Heatmap colours from light blue to dark blue indicate that IceNet outperformed the benchmark model in the majority of years. Data from Oct 2020-Dec 2020 was not used in this study, so the maximum number of years is 8 for Oct-Dec.

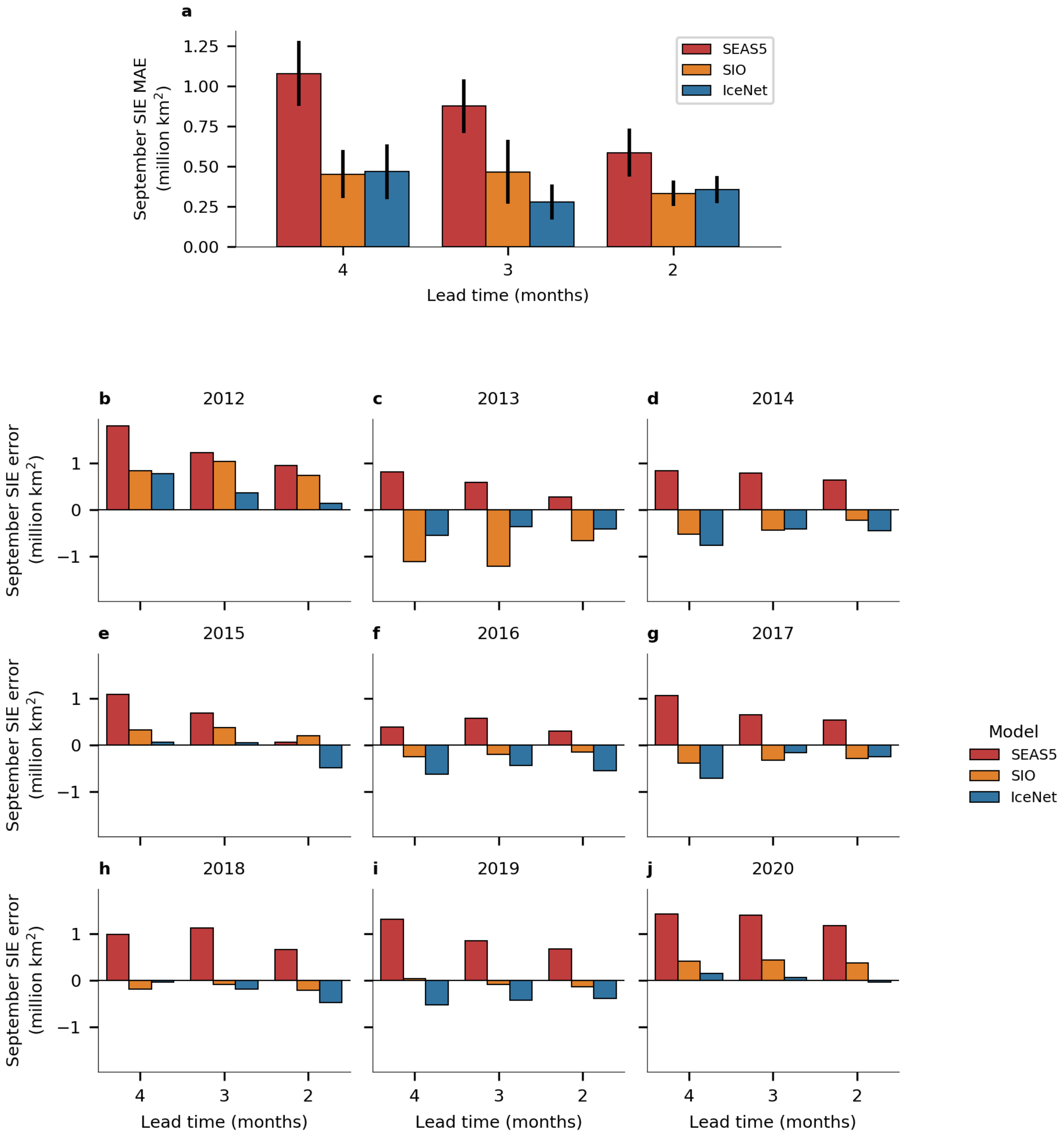

Supplementary Fig. 2 | **Comparison between IceNet, SEAS5, and the Sea Ice Outlook (SIO) ensemble median in predicting September sea ice extent (SIE). a**, Mean absolute error (MAE) over 2012-2020 versus lead time, with error bars showing one standard deviation. **b-j**, Year-wise breakdown. For IceNet and SEAS5 the SIE error is computed relative to the SIE of the monthly-mean OSI-SAF sea ice concentration data (see Methods), whereas for the SIO it is computed relative to the NSIDC Sea Ice Index.

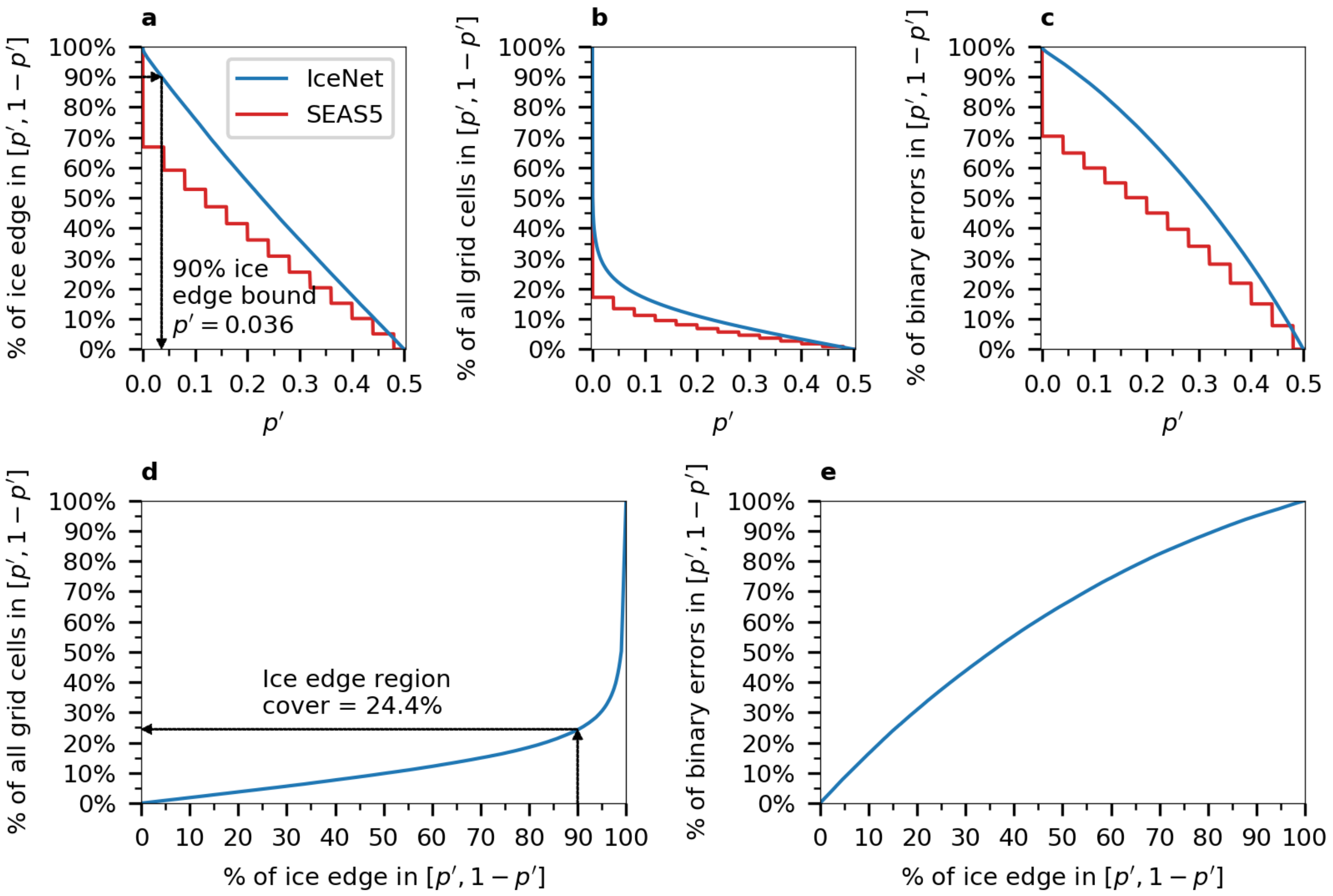

Supplementary Fig. 3 | **Determining the ice edge region: a probabilistic framework for bounding the ice edge. a**, Percentage of the observed ice edge contour contained within the forecast sea ice probability (SIP) bounds [p', 1 - p'] as a function of p', computed over the validation years (2012-2017) and all six lead times, shown for IceNet and SEAS5. SEAS5's curve, shown in red, has discontinuities because of its discretised SIP values. The arrow reads off the p' corresponding to 90% ice edge bounding, $p'_{90\%} = 0.036$, used to determine IceNet's ice edge region. **b**, The percentage of all grid cells bounded by [p', 1 - p'], relating to forecast sharpness, determining the size of the ice edge region. **c**, The percentage of binary errors bounded by [p', 1 - p']. **d**, Trade-off between ice edge bounding percentage and the size of the ice edge region. Bounding 90% of the ice edge with IceNet corresponds to labelling 24.4% of grid cells as the ice edge region. **e**, Relationship between the ice edge bounding percentage and the binary error bounding percentage.

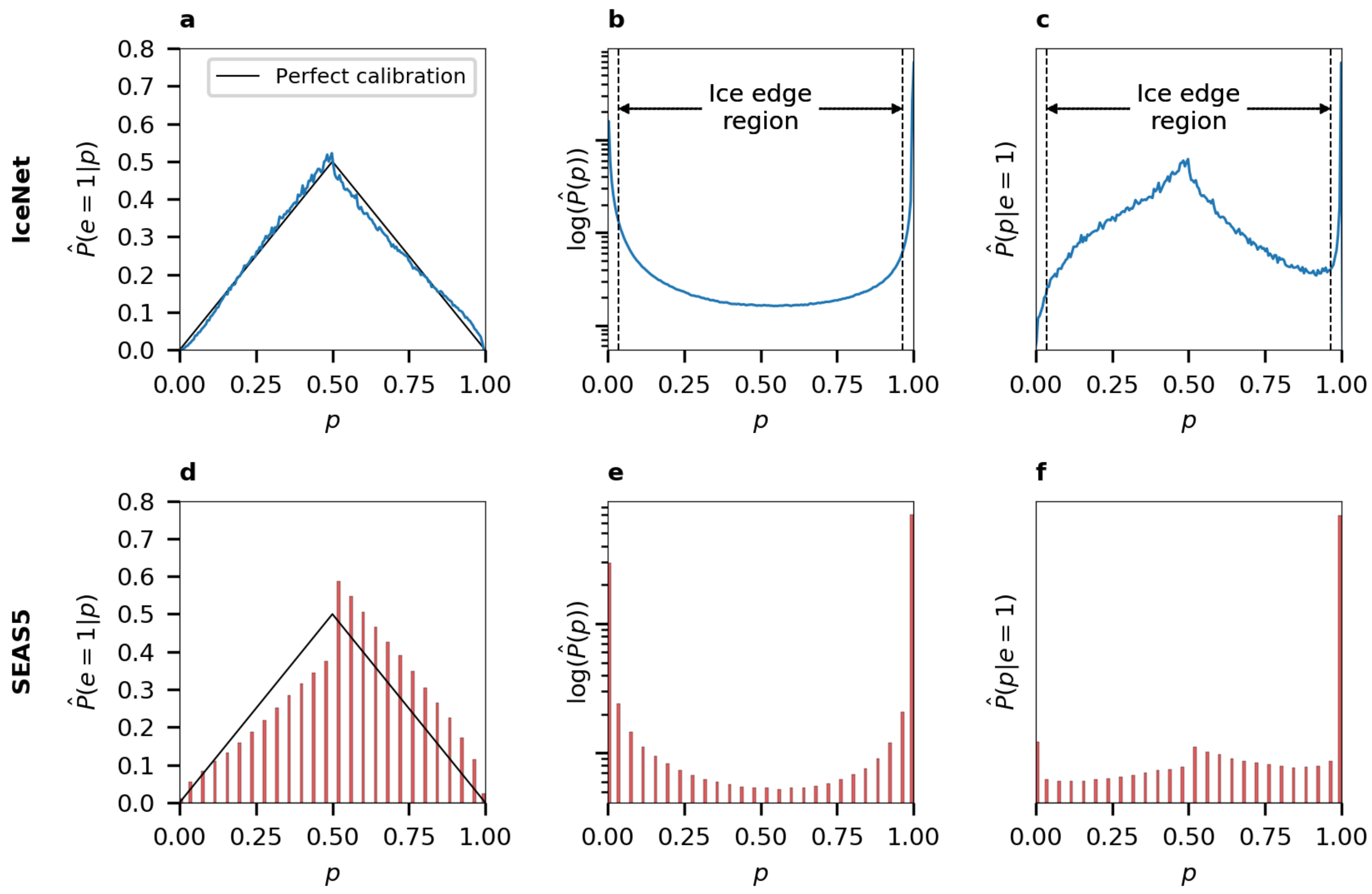

Supplementary Fig. 4 | **Relating the ice edge region to forecast calibration and sharpness. a-c**, IceNet's empirical probability densities for the sea ice probability (SIP), p, and a binary error variable e, where $e = 1$ if a binary error occurred and $e = 0$ if the correct class was predicted. The three distributions are related through Bayes' Rule: $P(p|e = 1) \propto P(e = 1|p) \cdot P(p)$. The distributions were computed over validation years 2012-2017 and all six lead times. **d-f**, The same empirical densities as **a-c** but for SEAS5. SEAS5 outputs discrete values for p so the distributions shown are discrete. **a, d**, Empirical binary probability of the binary error variable, e, given the sea ice probability p. A perfectly calibrated model whose p is equal to the actual frequency of sea ice will make errors according to the triangular curve overlaid. **b, e**, Empirical distributions of p over all grid cells, $P(p)$, measuring the forecast sharpness. $P(p)$ is dominated by peaks at $p = 0$ and $p = 1$ due to the many grid cells where ice or ocean always occur and prediction uncertainty is low. We plot the base-10 logarithm of $P(p)$ to highlight smaller values at intermediate values of p. **c, f**, Empirical distributions of p at grid cells where a binary error occurred, $e = 1$. The distributions in **a-c** were plotted using kernel density estimation with Gaussian kernels of bandwidth 0.005. The distributions in **d-f** were plotted using histograms with 99 equal-width bins in (0, 1).

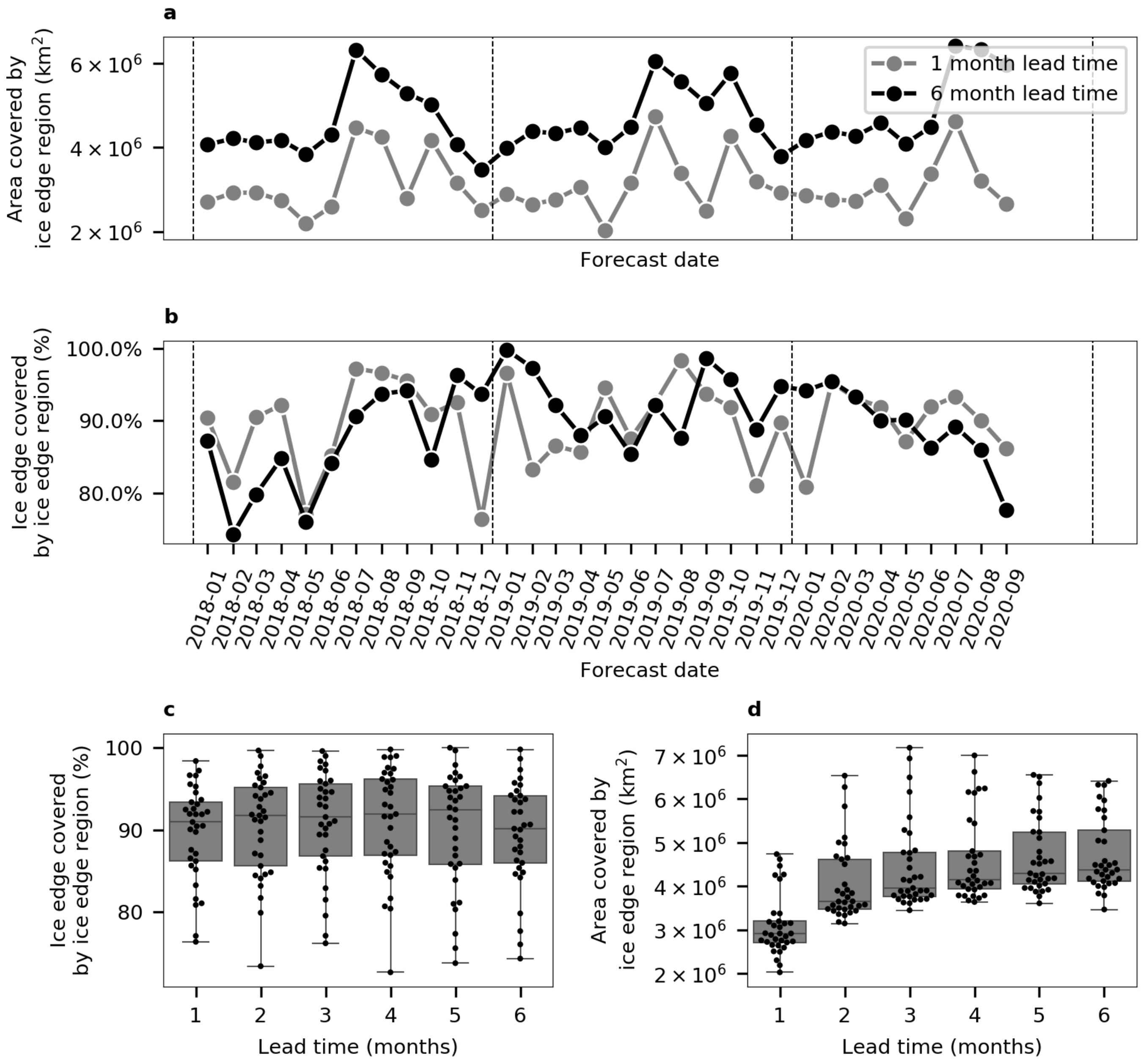

Supplementary Fig. 5 | **Evaluation of IceNet's ice edge bounding ability over the test years 2018-2020. a**, Area of IceNet's ice edge region versus forecast date for a 1-month and 6-month lead time. The size of the ice edge region increases when IceNet is more uncertain about the position of the ice edge, such as in summer or at long lead times. **b**, As above, but for the fraction of ice edge contour grid cells covered by the ice edge region. This fraction measures how well the ice edge region bounds the observed ice edge for a given forecast month. **c, d**, box-and-whisker plots for the ice edge coverage and area of the ice edge region versus lead time, with the individual samples overlaid as swarm plots. **c** shows that the average ability to bound the ice edge is roughly constant with lead time. **d** makes clear the increase in size of the ice edge region with lead time, although the strong seasonal variation within each lead time is masked (see Supplementary Fig. 6). For the box-and-whisker plots, the boxes show the upper quartile, median, and lower quartile, while the whiskers indicate the range of data values.

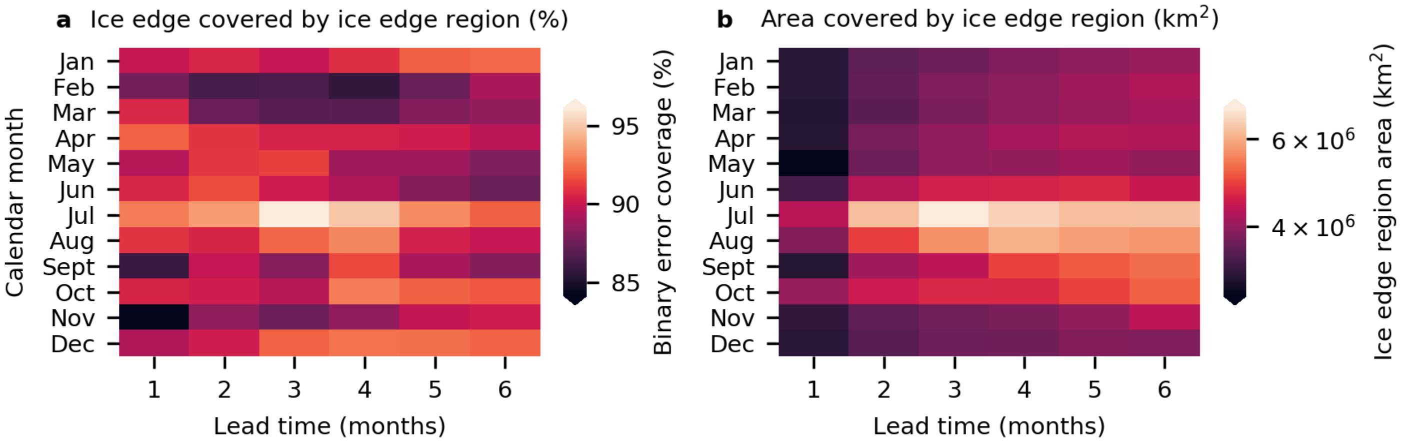

Supplementary Fig. 6 | **Effect of season and lead time on IceNet's ice edge region. a**, Heatmap of the percentage of the ice edge occurring within IceNet's ice edge region. There is not a clear dependence on season or lead time. **b**, As above, but for the area of the ice edge region, measuring uncertainty in the position of the ice edge. Forecasts that pass through the spring predictability barrier are linked to large uncertainty in the ice edge position. The values plotted are averages over both validation and test years, 2012-2020, to maximise the number of samples used.

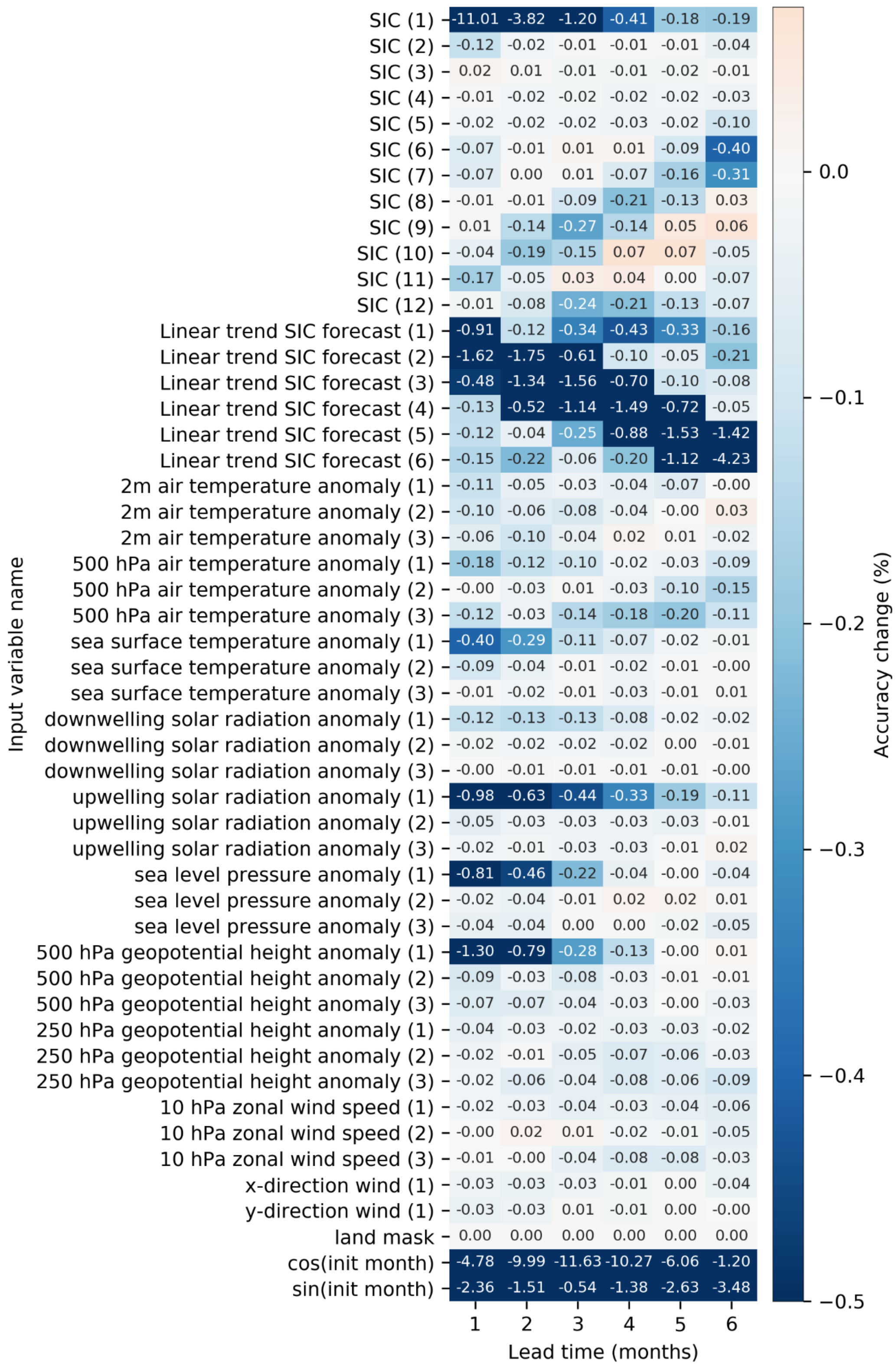

Supplementary Fig. 7 | **Full variable importance results from the permute-and-predict method.** Heatmap showing the mean accuracy change from the permute-and-predict method for each input variable and lead time, averaged over 10 random seeds and 96 forecast months (2012-2019). The number in brackets by the variable names indicate the input lag (or for the linear trend forecast input, the lead) in months. The colorbar is artificially saturated at -0.5% to emphasise the patterns at smaller magnitudes. SIC = sea ice concentration.

Supplementary Methods

Here we describe the pemute-and-predict method used to rank IceNet's input variables in terms of importance for its sea ice forecasts. The permute-and-predict method randomly permutes (i.e., shuffles) the 2D input fields of a particular input variable, keeping all other input variables at their standard values. For example, permuting the 1-month lag sea ice concentration (SIC) input variable may shift the August 2012 SIC input for a September 2012 forecast initialisation to the place of January 2016 SIC for a February 2016 forecast initialisation, repeating until each month of data for that variable has been permuted. This process cuts the ties between the input variable and the outputs. After permuting, IceNet is re-run over 2012-2019 using the permuted input dataset and the drop in three-class accuracy is measured (2020 data had not been downloaded at the time of running the method and is not used here). By repeating for each input variable, a full ranking is obtained for each forecast month and lead time. Although the accuracy drop can be averaged across each forecast month and lead time to produce a single ranking, this smears seasonal information due to the substantial changes in physical processes driving sea ice evolution in different calendar months and at different timescales. We thus employ a more instructive approach of separating the rankings by calendar month and lead time, producing 72 rankings (12 calendar months x 6 lead times). The accuracy drop was computed relative to IceNet's ensemble-mean forecasts in order to reduce noise associated with individual network input-output mappings.

Due to the random nature of permutation, different rankings may be obtained using different permutations; we repeat the method 10 times with varying random seed and compute the mean drop in accuracy to reduce random noise. Each accuracy drop in Table 1a and Table 1b is the mean of 80 values (10 random seeds x 8 years). A single repetition of the permute-and-predict method requires running IceNet 50 times over 2012-2019 (once for each input variable), and hence the computational cost scales linearly with the number of random seeds used for the averaging. To reduce this cost, we pruned the IceNet ensemble to 5 out of its 25 ensemble members. We chose 5 of the best-performing ensemble members in terms of accuracy over the validation years 2012-2017. By removing the IceNet ensemble members with a lower predictive capability, the

rankings produced are less likely to reflect spurious variable associations that weaker ensemble members might have learned.

The input variables 'cosine of initialisation month index' and 'sine of initialisation month index' (Supplementary Table 2) often correspond to large accuracy drops when permuted (Supplementary Fig. 7). We chose to remove these variables from the rankings in Table 1, as they only provide information about where in the annual cycle the forecast is initialised, and thus do not have a physical meaning.

The full permute-and-predict results reported in Supplementary Figure 7 show the accuracy drop values averaged over all calendar months. Therefore, each mean accuracy drop shown is the mean over N_{seeds} x N_{years} x 12 = 10 x 8 x 12 = 960 values.

Most variable importance methods that rank the importance of variables in statistical models have caveats associated with them. The permute-and-predict method has the limitation that correlated input variables can be assigned inflated importance[1]. This is because permuting an input variable that correlates with another can push the input data outside of the space where the network has seen training examples (and thus into a space where the input-output mapping is poorly defined). As a result of this potential importance inflation, the importance values in Table 1 should be considered as upper bounds on the true importance and interpreted with a degree of caution. However, we note that by removing the seasonal signals of the non-SIC input variables with seasonal cycles, the greatest source of correlation for these variables (that of autocorrelation with itself at different lags) is removed. Furthermore, despite the SIC input variables having strong autocorrelation due to the annual sea ice cycle, the accuracy drops for SIC at lags greater than 1 month were often very small (Supplementary Fig. 7), suggesting the importance inflation effect is small.

Supplementary Information References

1. Hooker, G. & Mentch, L. Please Stop Permuting Features: An Explanation and Alternatives. *ArXiv E-Prints* **1905**, arXiv:1905.03151 (2019).
